# Rheotaxis in Larval Zebrafish Is Mediated by Lateral Line Mechanosensory Hair Cells

**DOI:** 10.1371/journal.pone.0029727

**Published:** 2012-02-16

**Authors:** Arminda Suli, Glen M. Watson, Edwin W. Rubel, David W. Raible

**Affiliations:** 1 Department of Biological Structure, University of Washington, Seattle, Washington, United States of America; 2 Department of Biology, University of Louisiana Lafayette, Lafayette, Louisiana, United States of America; 3 V. M. Bloedel Hearing Research Center, University of Washington, Seattle, Washington, United States of America; Texas A&M University, United States of America

## Abstract

The lateral line sensory system, found in fish and amphibians, is used in prey detection, predator avoidance and schooling behavior. This system includes cell clusters, called superficial neuromasts, located on the surface of head and trunk of developing larvae. Mechanosensory hair cells in the center of each neuromast respond to disturbances in the water and convey information to the brain via the lateral line ganglia. The convenient location of mechanosensory hair cells on the body surface has made the lateral line a valuable system in which to study hair cell damage and regeneration. One way to measure hair cell survival and recovery is to assay behaviors that depend on their function. We built a system in which orientation against constant water flow, positive rheotaxis, can be quantitatively assessed. We found that zebrafish larvae perform positive rheotaxis and that, similar to adult fish, larvae use both visual and lateral line input to perform this behavior. Disruption or damage of hair cells in the absence of vision leads to a marked decrease in rheotaxis that recovers upon hair cell repair or regeneration.

## Introduction

Mechanosensory hair cells are specialized cells with protruding apical stereocilia whose deflection leads to voltage changes that are transmitted to the central nervous system. The auditory system, vestibular system and mechanosensory lateral line system utilize these sensory cells as the first stage of processing information about the environment. The lateral line system, present in fish and amphibians, allows these organisms to respond to mechanical stimuli generated from water motion in their surroundings. This system is composed of sensory organs named neuromasts, which are located in canals or deposited on the body surface of the developing larvae [1], [2]. The mechanosensory hair cells reside in the center of neuromasts and are surrounded by interdigitating glial-like support cells [2], [3]. Behaviors such as schooling [4], prey detection [5], [6] and escape [7], [8] have been shown to be dependent on the proper function of the lateral line system.

Unlike their mammalian counterparts, hair cells of birds [9], [10], amphibians [3], [11] and fish [12], [13], [14], [15] regenerate following cell damage [16]. The easily accessible mechanosensory lateral line has emerged as an excellent system in which to study hair cell death and regeneration in the hopes of understanding the mechanisms underlying these processes and identifying potential therapeutic solutions for hearing and balance disorders. Since lateral line mechanosensory hair cells are able to regenerate, developing a functional assay following hair cell perturbation is of particular interest.

In adult fish, rheotaxis, the ability to align against current, partially depends on the lateral line system [17], [18], [19]. While there is evidence that larval zebrafish can swim under flow conditions [20], [21], it is not known to what extent larval rheotaxis depends on lateral line function. To address this issue, we developed methods to evaluate rheotaxis under constant flow conditions. We then varied flow rate, lighting conditions and presence, damage, absence or regeneration of lateral line hair cells and tested their effect on rheotaxis. Of most interest, we report that of hair cells or damage to stereocilia bundle integrity disrupts rheotaxis, and that normal rheotaxis resumes upon repair of bundle integrity or with regeneration of hair cells.

## Methods

### Ethics statement

All experiments were approved by the University of Washington Institutional Animal Care and Use Committee, AWA number A3464-01.

### Rheotaxis apparatus and assay

**Figure 1:**
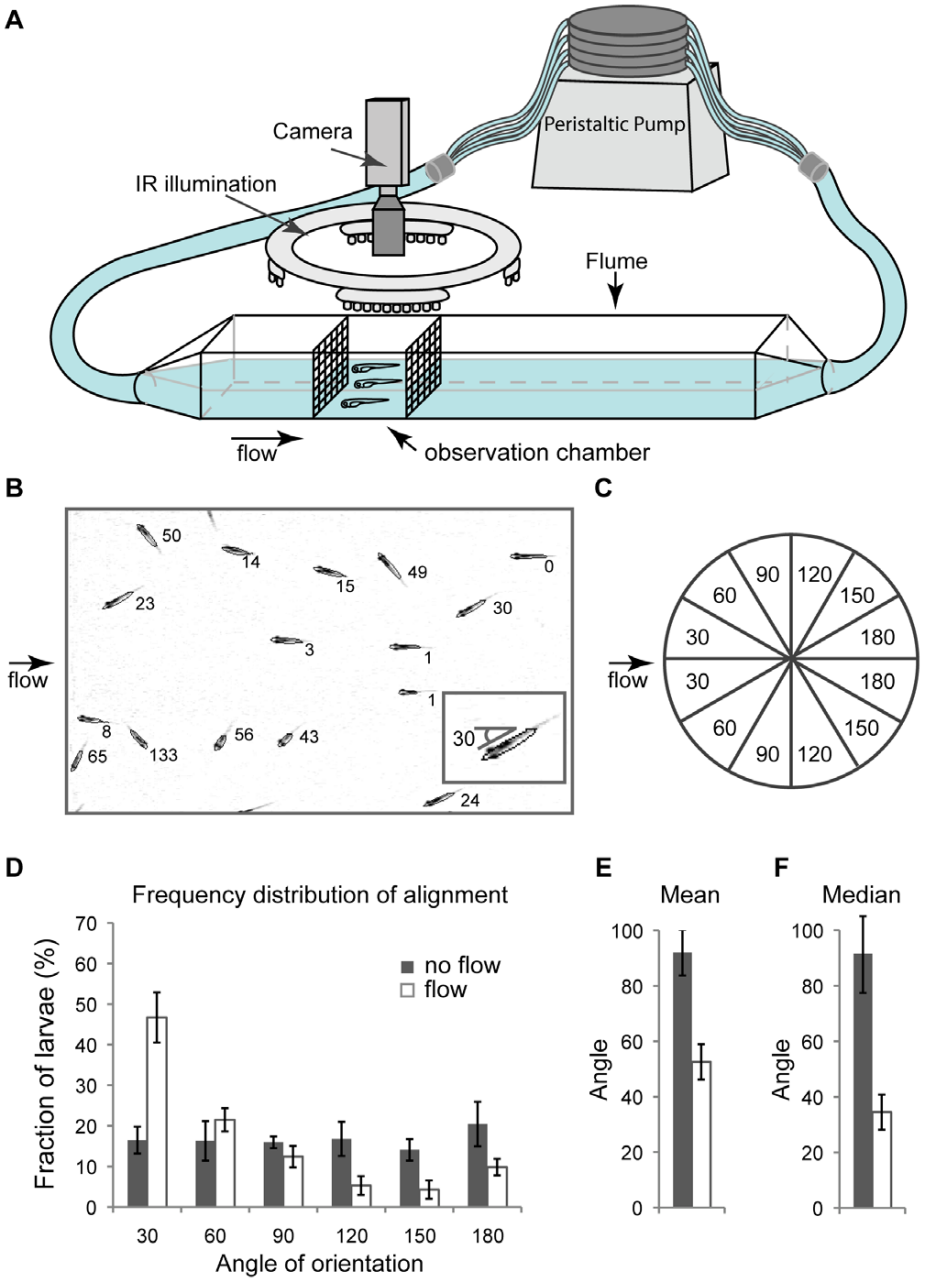
Zebrafish larvae can rheotax in flow conditions. A) We built a clear plastic, rectangular flume in which to test rheotaxis. 20–30 larvae are placed in observation chamber and their alignment is recorded. B) Using ImageJ (NIH) macros, images are cropped, ellipses are fitted around each larva, and their alignment angle against current is calculated. Larvae display a preference for aligning against current in flow conditions (0.14 cm/s) as shown by frequency distribution (D), mean (E) and median (F) of alignment. C) Larvae show no left-right preference; therefore, their alignment is calculated in a 0–180° scale (C). Experiment performed in IR.

A 1.1 m length×3.7 cm width×5.1 cm height clear plastic flume was connected to a peristaltic pump (Dynamax, RP-1, Rainin,) to create a closed flow system (Figure 1A). The flume was filled with E2 (embryo medium) [22] to a depth of 2.8 cm, and the pump generated flow up to 0.2 cm/s. Two stainless steel screens were placed 5 cm apart toward the middle of the flume, partitioning an observation area. We initially placed drinking straws upstream of the observation chamber to act as collimators, but found that the screens were sufficient to create laminar flow within the observation area. To test for laminar flow, we applied 2% phenol red upstream of the first screen and imaged dye movement from the side. We observed a boundary layer effect in the bottom 0.5 cm of the flume that caused no turbulence to the rest of the column. A Dage-MTI (Michigan City, IN) CCD72 camera was placed above the observation chamber for imaging. A water heater (Visi-therm 25 W, Aquarium Systems, Mentor, OH) was placed downstream of the observation chamber to maintain temperature of re-circulating medium between 26–28°C. In experiments in which we sought to eliminate visual input to larval, we captured images under infrared illumination, lighting the chamber from above with an IR LED light array (920 nm IR-TILE, PolarisUSA, Norcross, GA).

We used the AB strain in all experiments. Embryos were collected and kept at 28°C in a 14 hr/10 hr light/dark cycle. Larvae were assayed at 5 days post fertilization (dpf), when they reached ∼0.33 cm in length. 5 dpf larvae were dark-adapted for at least 30 min (EDTA experiments) or 3–4 hr (all other experiments) prior to testing. All experiments were performed at the same time of day since larval locomotor activity is affected by circadian rhythms [23], [24]. For each experiment, 20–30 larvae were placed in the observation chamber, allowed to explore their new environment for 3 min and then imaged 10 times, 10 sec apart, to determine alignment under no-flow condition. The pump was then turned on, and larvae swam for 3 min before another set of 10 images, 10 sec apart, was acquired. No larvae experienced flow before being tested, except in the EDTA treatment and the regeneration experiments. For EDTA experiments, larvae were tested both acutely (from 30–90 min post treatment), and after recovery (from 4–5 hr hours post treatment (hpt)). For regeneration experiments, larvae were tested at 4–5 hpt and again at 52–53 hpt, enough time for hair cell regeneration to occur [12], [25].

### Image analysis

BTVPro (BenSoftware) was used to control the camera and capture images (Figure 1A,B). ImageJ (NIH) was used for analysis. We initially cropped images to exclude the 0.5 cm boundary layer, but found that results were not changed when this area was included in calculations. Subsequently, images were cropped only to the edges of the observation area. A background subtraction macro was applied to smooth any differences in background illumination. A threshold macro recognized larvae in the image, and an ellipse was fitted around selected objects (Figure 1B). A line drawn from the center of the ellipse to the center of mass of each object resulted in a vector representing the long axis of larvae. This vector was compared to flow direction to provide an alignment angle for each larva. We manually checked the alignment angle of more than 600 larvae and found that this macro assigned angles appropriately. Larvae crossing each other or touching the sides of the cropped image were excluded from analysis. Larvae that did not change their position in subsequent images were presumed to be stuck at the bottom of flume and were excluded; these represented a small fraction (3–4%) of all larvae.

After determining the alignment of larvae in each frame, data were pooled across the 10 frames of a single experiment, and the frequency distribution of the alignment with respect to flow was calculated. Angles of alignment were represented in a 0–180° scale, since larvae showed no left-right preference of alignment. Each experiment was repeated 4–5 times with a different group of larvae and the mean of means was plotted. All the data we present shows this mean of means +/− 1 SD.

### Drug treatments

Lateral line hair cells were killed by incubating the larvae for 1 hr with 50–400 µM neomycin (Sigma, St Louis, MO) [12]. Larvae were incubated with drug in the dark at 28°C. The effectiveness of hair cell death was monitored by incubation with 0.005% DASPI stain after the rheotaxis assay was completed. To break hair cell tip-links, we incubated the larvae for 20 min in 1 mM EDTA in embryo medium [26]. To confirm the effectiveness of treatment we treated larvae with 150 µM FM1–43 (Invitrogen, Carlsbad, CA) for 1 min and then assessed dye uptake by hair cells [27], [28], [29].

## Results

Five day-old zebrafish larvae were tested for rheotaxis in continuous, non-turbulent flow. At this stage of development, lateral line hair cells are functional as shown by physiological recordings, vital dye uptake and their susceptibility to damage by aminoglycosides [30], [31]. We placed larvae in a flow of 0.14 cm/s, and measured their cumulative overall alignment 3 min after flow onset (Figure 1D). In the absence of flow, larvae show random alignment with respect to the water entry point, with mean and median alignments close to 90° (Figure 1E,F, black bars). By contrast, in the presence of flow, the overall mean and median alignments are significantly closer to the opposite direction of flow (Figure 1E,F, open bars). Under flow conditions, at any given time larvae are engaged in several different behaviors, including rheotaxis, turning or directed swimming (Movie S1). These different behaviors are apparent when the distribution of orientation angles is examined (Figure 1D). About half of larvae are oriented within 30° of the direction of flow and about 90% are oriented within 90°, whereas under no-flow conditions no bias for directions exists (Figure 1D).

**Figure 2:**
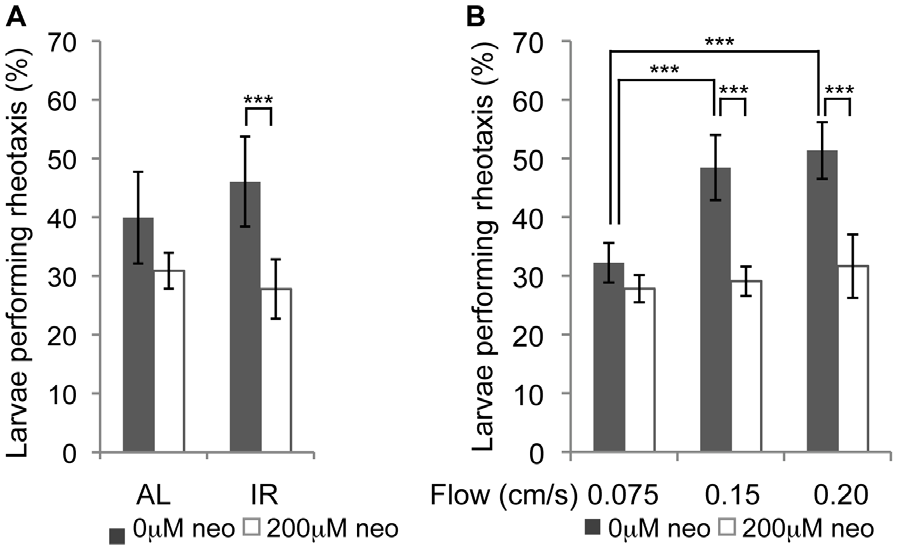
Zebrafish larvae use the lateral line system to align in flow. Alignment of larvae at 30° is shown. A) Removing visual cues and lateral line input leads to reduced rheotaxis (n = 5) (flow = 0.16 cm/s). AL: ambient light illumination; sides of flume covered by paper with vertical stripes; IR: infrared illumination. B) Untreated larvae align better with increased flow rates, while alignment of lateral-line-impaired larvae does not change significantly at different flow rates (n = 5). Statistics: ANOVA with Tukey post-test. Error bars show standard deviation. *** p<0.001.

A first step in our analyses was to determine the optimum conditions for testing lateral line hair cell contribution to rheotaxis. Toward this end, we tested rheotaxis in different lighting conditions and various flow rates. In both cases, the importance of lateral line hair cells was assessed by acute destruction of functional lateral line hair cells with neomycin exposure [12], [31]. Both visual and lateral line input have been shown to play a role in rheotaxis in adult fish [18], [19], [32]. To determine the relative importance of these stimuli for zebrafish larval behavior, we tested rheotaxis in both ambient light (AL) and infrared light (IR) in untreated controls and after ablation of lateral line hair cells with 200µM neomycin (Figure 2A). Bath-application of neomycin does not kill auditory and vestibular hair cells or lead to balance phenotypes (data not shown), therefore, we do not expect the auditory and vestibular systems to be affected. To present the larvae with controlled visual cues we covered the sides of our clear flume with a vertically striped black and white paper (1 cm apart). To statistically distinguish among different conditions, we repeated each experiment 6 times on different days and compared the mean statistics, since the day-to-day variation proved to be greater than within-day variation. For all experiments we found that comparing means, medians or frequency of alignment within 30° of the flow did not change the outcome of statistical analysis. Hence, the rest of the results are reported as frequency of alignment within 30°, which we define as rheotaxis. When we removed lateral line input and tested larvae in AL, fewer larvae undergo rheotaxis, although this difference is not statistically significant (Figure 2A). When we removed visual input (IR condition) in addition to lateral line input, we found that the frequency of rheotaxis of treated larvae decreased significantly when compared to untreated controls. These results suggest that removing inputs from both the lateral line and the visual system is more detrimental to rheotaxis than removing the lateral line input alone. It is interesting to note that the trend of alignment in untreated larvae is less in AL then in IR illumination (although not significant), suggesting that other directed movements in these conditions may obscure the measurement of rheotaxis. Since significant differences in rheotaxis were observed only in IR, all subsequent experiments were performed under IR illumination.

To determine if flow velocity affects the likelihood of larvae to perform rheotaxis, we exposed control and neomycin-treated larvae to different rates of flow, specifically: 0.075 cm/s, 0.15 cm/s, and 0.2 cm/s. We found that alignment improves for untreated larvae when we increase the flow speed from 0.075 cm/s to 0.2 cm/s (Figure 2B). Alignment did not improve significantly when the flow speeds were increased beyond 0.15 cm/s, suggesting the existence of an upper limit for the larval lateral line input. In contrast, changing the flow rate had no significant effect on rheotaxis after lateral line damage. All further studies were conducted in flow rates of 0.14–0.16 cm/s.

**Figure 3:**
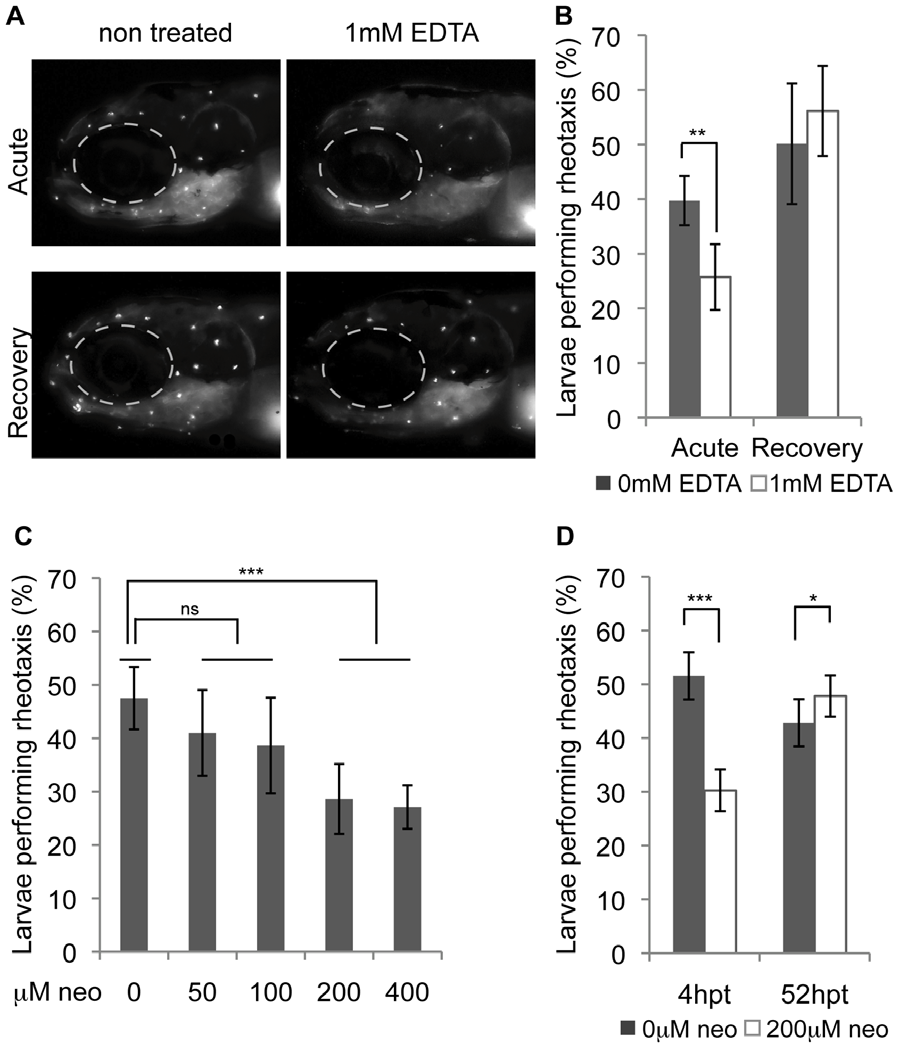
Rheotaxis depends on level of hair cell impairment or damage and is restored during regeneration. A) Mechanotransduction, as shown by FM1–43 dye uptake, is blocked when tip-links are broken at ∼30 min after EDTA treatment (acute) and is restored again at 4–5 hours post treatment (recovery) (flow = 0.16 cm/s). B) Rheotaxis is impaired acutely after EDTA treatment and restored to control levels after recovery (n = 5). C) Ablating the lateral line input by different neomycin doses leads to a dose dependent rheotaxis impairment (n = 6) (flow = 0.14 cm/s). D) Rheotaxis is restored during mechanosensory hair cell regeneration (n = 5) (flow = 0.16 cm/s). Statistics: C) Statistics: ANOVA with Tukey post-tests. B,D) Paired t-test. Error bars show standard deviation. * p = 0.01–0.05, ** p = 0.01–0.001, *** p<0.001.

To further assess the relationship between lateral line hair cell function and rheotaxis, we treated the embryos with a 1 mM solution of the calcium chelator, EDTA, which presumably breaks tip-links and therefore disrupts mechanotransduction, but does not destroy the hair cells. Other calcium chelators have been shown to break hair cell tip-links in order to disrupt mechanotransduction [33], [34], and significantly reduce rheotaxis in adult blind cave fish [26]. To assess whether treatment with EDTA blocked mechanotransduction, we assayed FM1–43 uptake, a fluorescent dye that accumulates in hair cells in a mechanotransduction-dependent manner [27], [28], [29]. When we applied FM1–43, 30 min after washing out the EDTA, we found that its uptake was largely inhibited (Figure 3A). However, when this procedure was repeated at 4–5 hpt (hours post EDTA treatment), we saw that FM1–43 uptake was restored to qualitatively normal levels, suggesting that tip-links are restored by this time. We found similar effects on rheotaxis. Significantly fewer larvae performed rheotaxis at 30–90 min after EDTA treatment, but rheotaxis had fully recovered at 4–5 hpt (Figure 3B). We do not believe that EDTA treatment affects the hair cells of inner ear sensory patches since no circling or balance behaviors were observed. However, we were not able to confirm this by FM1–43 dye uptake since the inner ear hair cells are not directly exposed to the embryo medium. Given that EDTA treatment recapitulates the effect of neomycin on rheotaxis, we feel confident that lateral line hair cell activity is important for rheotaxis in the zebrafish larvae.

We next tested whether differential damage to the lateral line system is reflected in systematic changes in rheotaxis. Lateral line hair cells are ablated by aminoglycoside antibiotics in a dose-dependent manner, with little damage occurring at 50 µM and almost complete damage at 200µM [12], [31], [35]. We find a dose-dependent change in rheotaxis, with decreasing alignment as the dose is increased (Figure 3C). As previously demonstrated for hair cell loss, decreases in rheotaxis reached a maximum at 200µM (Movie S2), showing little differences at higher concentrations. These experiments provide evidence that hair cell damage leads to a significant decrease in the ability to orient against flow in 5 dpf zebrafish larvae, therefore, indicating that lateral line input is important for flow detection as early as the initial stages of larval development.

Since both neomycin and EDTA treatments implicate lateral line hair cells in rheotaxis, we wanted to know if this behavior is restored when hair cells regenerate. Two days after neomycin-induced damage, the majority, but not all, of hair cells have regenerated and neurons have made contacts [25], [36], [37]. When we ablate hair cells using neomycin and assay rheotaxis at 4–5 hpt, we find that the larvae's ability to align against flow is significantly reduced (Figure 3D). However, at 52–53 hpt the treated larvae display near-normal rheotaxis. We conclude that rheotaxis is restored following hair cell regeneration.

## Discussion

Rheotaxis, defined as “alignment in a stream”, is one of several behaviors employed by fish to position themselves in an appropriate position for catching food floating downstream, for migration, for reaching spawning sites, etc (reviewed by Arnold [17]). Similar to previous observations [20], [21], our results show that zebrafish larvae can align by positive rheotaxis (i.e., aligning to face the oncoming flow) under moderate flow conditions. In their natural habitat, zebrafish spawn in areas of low flow [38], but we do not know if zebrafish larvae perform rheotaxis under natural conditions. Nevertheless, the fact that larvae are capable of performing positive rheotaxis demonstrates that the sensory and motor systems responsible for this behavior are in place and functional at larval stages.

Early experiments by Lyon [32] attributed rheotaxis of fish mainly to the visual system, suggesting that fish orient to movements across the visual field. Orientation to movement of stimuli across the visual field results in optomotor behavior in zebrafish larvae, where fish swim in the direction of perceived motion [39]. Montgomery and colleagues [19] showed that lateral line of adult fish is also critical for orientation. Likewise, we find that in 5 dpf zebrafish larvae, the visual system and the lateral line system are important for rheotaxis. At an intermediate flow rate, we find that vision and lateral line can compensate for each other during rheotaxis when either input is inhibited. However, when inputs from both systems are removed rheotaxis is greatly reduced. Montgomery and colleagues [19] attribute the input from the superficial neuromasts and not canal neuromasts to be responsible for the lateral line dependent rheotaxis. Superficial neuromasts in torrent fish, Antarctic fish, and blind cavefish are activated at lower flow rates (up to 1 body lengths per second (bl/s), 0.25 bl/s, and 1.6 bl/s respectively), while canal neuromasts are activated at higher flow (1.7 bl/s, 0.4 bl/s, and 2.9 bl/s respectively). In 5 dpf larval zebrafish only superficial neuromast are developed. We find that at flow rates up to 0.6 bl/s lack of hair cells in superficial neuromasts leads to a decrease in rheotaxis, which is consistent with findings in adults.

Despite the considerable reduction in rheotaxis, when both the lateral line and visual inputs are eliminated, the larvae still display significant alignment, suggesting that input from other sensory systems must feed into the rheotaxis circuitry. Lyon and Montgomery [19], [32] suggested that somatic inputs are also important for rheotaxis. We observed that larvae come in contact with the side or bottom of the chamber, also suggesting that they may use touch for orientation. Whether larvae are more likely to use touch for orientation after lateral line damage would require additional monitors to track behavior in several dimensions. Fish can also use chemotaxis in combination with the lateral line system for additional guidance cues [18]. Performing the rheotaxis assay in an open system as opposed to the re-circulating system, showed no statistical difference between the two (data not shown). Therefore, the potential release of odorants by the larvae in the re-circulating system was not sufficient to affect rheotaxis. It would be interesting to see if adding other odorants can increase the percentage of larvae aligning against current at any given time.

We found that regeneration of lateral line hair cells restores rheotaxis perturbed by neomycin treatment. Rheotaxis was restored to near normal levels by 48–52 hrs after neomycin exposure, even though the hair cell regeneration was incomplete at this time [25], suggesting that perhaps there is a threshold of input needed from the lateral line above which the behavior does not change. Similar results were found by McHenry et al. [40], who measured lateral line-mediated escape response. A full complement of hair cells within lateral line organs may provide sensitivity to stimuli not measurable under either assay.

Our work has established a behavioral assay by which we can assess lateral line function. Because lateral line hair cells are situated on the body surface during larval development, zebrafish have proved to be an accessible model for investigating hair cell death, protection, regeneration, and synaptogenesis [12], [13], [14], [15], [35], [36], [37], [41], [42], [43], [44]. Studying these mechanisms in such an accessible model could potentially shed light on similar processes of other types of hair cells, namely those found in the auditory and vestibular systems, which are not as amenable to perturbations and *in vivo* analyses. Finally, our assay could be beneficial in isolating functional mutants that affect not only the mechanosensory hair cells but their neuronal circuitry as well.

## Supporting Information

Movie S1 **5 day-old zebrafish larvae perform rheotaxis.** Larvae orient when placed in a flow of 0.16 cm/s. Larvae are engaged in several behaviors, including rheotaxis, turning and directed swimming.
(MOV)

Movie S2 **Perturbation of lateral line hair cells decreases the ability of 5 day-old zebrafish larvae to perform rheotaxis.** After treatment with 200 µM neomycin, larvae show decreased rheotaxis while still engaging in other behaviors.
(MOV)
